# Generation of Human *CEACAM1* Transgenic Mice and Binding of *Neisseria* Opa Protein to Their Neutrophils

**DOI:** 10.1371/journal.pone.0010067

**Published:** 2010-04-09

**Authors:** Angel Gu, Zhifang Zhang, Nan Zhang, Walter Tsark, John E. Shively

**Affiliations:** 1 Department of Immunology, Beckman Research Institute of the City of Hope, Duarte, California, United States of America; 2 Department of Biology, Beckman Research Institute of the City of Hope, Duarte, California, United States of America; Columbia University, United States of America

## Abstract

**Background:**

Human CEACAM1 is a cell-cell adhesion molecule with multiple functions including insulin clearance in the liver, vasculogenesis in endothelial cells, lumen formation in the mammary gland, and binding of certain human pathogens.

**Principal Findings:**

Three genomic BAC clones containing the human *CEACAM1* gene were microinjected into pronuclei of fertilized FVB mouse oocytes. The embryos were implanted in the oviducts of pseudopregnant females and allowed to develop to term. DNA from newborn mice was evaluated by PCR for the presence of the human *CEACAM1* gene. Feces of the PCR positive offspring screened for expression of human CEACAM1. Using this assay, one out of five PCR positive lines was positive for human CEACAM1 expression and showed stable transmission to the F1 generation with the expected transmission frequency (0.5) for heterozygotes. Liver, lung, intestine, kidney, mammary gland, and prostate were strongly positive for the dual expression of both murine and human CEACAM1 and mimic that seen in human tissue. Peripheral blood and bone marrow granulocytes stained strongly for human CEACAM1 and bound *Neisseria* Opa proteins similar to that in human neutrophils.

**Conclusion:**

These transgenic animals may serve as a model for the binding of human pathogens to human CEACAM1.

## Introduction

CEACAM1 (carcinoembryonic antigen related cell adhesion molecule-1) is a type 1 transmembrane protein expressed on most epithelial cells, phagocytes and activated lymphocytes [1], [2]. In the case of epithelial cells, its cell adhesion activity is associated with diverse functions including angiogenesis [3], [4], lumen formation [5], [6], [7] and the binding of extracellular pathogens [8]. In the case of neutrophils, it may play a role in the binding of neutrophils to activated endothelium and extravasation into inflamed tissues [9], [10], and similar to epithelial cells, may bind extracellular pathogens [8], [11], [12]. In the case of activated lymphocytes, it serves as an inhibitory molecule, controlling the strength and duration of the immune response [2]. CEACAM1 is a critical molecule well situated to play a communication role between epithelial cells, that represent the first barrier to infection, and immune cells that control infections that disrupt the epithelial barrier. Thus, pathogens that bind to CEACAM1 may interfere with its normal function, namely maintenance of communication between these two barriers to infection.

CEACAM1 has multiple splice forms that are differentially expressed between epithelial and immune cells. The so-called “long form” has a cytoplasmic domain containing two ITIMs (immunoreceptor tyrosine-based activation motifs) that, when phosphorylated, may bind Src homology 2 domain-containing tyrosine phosphatases SHP1 and SHP2 [13] to convey inhibitory signals to the T-Cell receptor [14], [15], [16], B-Cell receptor [17], and epithelial growth factor receptor [18]. The long form also has a β-catenin binding site [19] and a filamin A binding site [20]. The so-called “short form” lacks the ITIMs, but has the ability, like the long form, to bind actin, calmodulin, and annexin II [21]. These activities indicate a strong association of CEACAM1 with actin cytoskeleton [7], [21], [22].

Among the human extracellular pathogens known to bind to human CEACAM1, *Neisseria gonorrhoeae* and *Neisseria meningitidis* have been shown to bind via the expression of their Opa (colony opacity-associated) proteins leading to adherence and invasion into epithelial cells [11], [23] and engulfment by neutrophils [8], [23], [24], as well as suppression of the T-lymphocyte response [25] and killing human B cells [26] effects that presumably inhibit antibody production. As mentioned above, the inhibitory activity is mediated by phosphorylation of the ITIMs on the long form of CEACAM1 and subsequent recruitment of SHP1/2 [13], [27] that can disrupt phosphorylation of important receptors, including the insulin receptor [28], EGFR [29], TCR [14], [15], BCR [17], and TLR2 [30]. In the case of *Neisseria gonorrhoeae*, neutrophils, due to their high levels of CEACAM1 expression, are a major target for spreading the infection in the urogenital tract. A major limitation to studying these interactions *in vivo* has been the lack of an animal model, since murine CEACAM1 is not a receptor for these human pathogens. In order to overcome this limitation, we have generated a human *CEACAM1* transgenic mouse that faithfully expresses human CEACAM1 in the expected tissues, including neutrophils. We demonstrate here that the neutrophils from the transgenic mice as well as human neutrophils bind gonococcal Opa_52_ protein expressed on *E. coli*. Thus, these mice should serve as an appropriate animal model at least for studying some aspects of the human pathogenesis of *Neisseria gonorrhoeae* infections.

## Materials and Methods

### Growth and isolation of Bacterial Artificial Chromosome (BAC)

Three BAC clones were purchased from Invitrogen. The DNA was cloned into the *Eco*RI sites of pBACe3.6 vector and transformed into DH10B™ *E. coli*. The vector contains a chloramphenicol resistance gene as the selectable marker. The BAC clone numbers and maps are given below: BAC#2: catalog#96012, Clone ID: 2023G5 Collection: BAC Human CTD, 214 kb, from 47,526867 to 47,741809, map: http://www.ncbi.nlm.nih.gov/projects/mapview/maps.cgi?TAXID=9606&CHR=19&MAPS=ideogr%2Ccntg-%2CugHs%2Cgenes&BEG=47526867&END=47741809&thmb=on BAC#3 catalog#96012, Clone ID: 2016N11. Collection: BAC Human CTD, 138 kb from 47,650836 to 47,788904, map: http://www.ncbi.nlm.nih.gov/projects/mapview/maps.cgi?TAXID=9606&CHR=19&MAPS=ideogr%2Ccntg-%2CugHs%2Cgenes&BEG=47650836&END=47788904&thmb=onBAC#5 catalog#96012, Clone ID: 2526I10 Collection: BAC Human CTD, 211 kb from 47,665,525 to 47,877001, map: http://www.ncbi.nlm.nih.gov/projects/mapview/maps.cgi?TAXID=9606&CHR=19&MAPS=ideogr%2Ccntg-%2CugHs%2Cgenes&BEG=47665525&END=47877001&thmb=on

The DNA of the BAC clones was extracted with Nucleobond PC 2000 EF kit (Macherey-Nagel, Düren Germany) according to the manufacturer's protocol. DNA pellets were dissolved in endotoxin-free water overnight at room temperature, and then used for microinjection.

### Chromosomal integration site analysis

Murine bone marrow cells were collected from femurs (see later section in Methods) and treated with colcemid (6 µl/mL in 0.075 M KCl) for 2 hours. The cells were fixed using Carnoy's fixative (3∶1; methanol:acetic acid), dropped onto non-silanized slides, and air-dried. The slides were baked at 60°C for 12 hours and aged at room temperature for 72 hours. Prior to banding cells were aged at 90°C for 15 minutes. Metaphases were GTG banded and photographed with the BandView imaging system from Applied Spectral Imaging (ASI). Upon completion of the GTG analysis, the slide was de-oiled in Americlear. Slides were de-stained in methanol for 5 minutes and rehydrated in an ethanol series: 100%, 80%, and 70% ethanol. Subsequently, the slides were post-fixed in 1% formaldehyde/1XPBS/50mM MgCl_2_ for 2 minutes at room temperature, washed in 1x PBS for 5 minutes and dehydrated in 70%, 80%, and 100% ethanol series. Once the slides were air-dried, they were denatured in 70% formamide/2x SSC at 72°C for 30 seconds and immediately placed in cold 70%, 80%, and 100% ethanol and air-dried. Denatured mouse SkyPaint probe (10 µl, Applied Spectral Imaging) was applied to each slide, covered with a glass coverslip and sealed with rubber cement. Slides were transferred to a humidified chamber and placed in a 37°C incubator for 48 hours. Post-washes and detection was done following ASI SKY protocol. SKY metaphases were captured and analyzed with the SkyVision spectral imaging system (Applied Spectral Imaging).

CEACAM1 BAC2 DNA was used as a probe. Three nick reactions were prepared using digoxigenin-dUTP. Reactions were combined for precipitation and re-suspended in 10 µl of 10 mM Tris buffer pH 8.5 for hybridization. The probe was hybridized to a human male metaphase to check for probe intensity and correct integration site. The probe (100 ng) was combined with 9 µL hybridization buffer, co-denatured on an 80° hotplate for 5 minutes and incubated overnight at 37°C in a humidified chamber. Post-wash consisted of 50% formamide/2x SSC at 45°C for 15 minutes, 2x SSC at 37°C for 8 minutes and 1x PBD at room temperature for 2 minutes. Probe was detected with rhodamine anti-digoxigenin and following SKY imaging, the slide was placed in 1x PBD for coverslip removal and immersed in room temperature 2x SSC for 1 hour to remove the SKY probe. After SSC treatment, slides were dehydrated and co-denatured for 3 minutes. Hybridization and post-wash were run as described above. Bone marrow preparations from a wild type mouse were used as a negative control and normal human male preparations were used as a positive control. Total murine bone marrow cells were prepared as described in a later section in Methods. Male bone marrow cells were discard tissue from the City of Hope bone marrow transplantation unit.

### Growth and FITC labeling of Opa_52_ *E. coli* and vector control *E.coli*

*E. coli* Top10 containing pTrc99A (Amp) vector only or pTrc99A vector with an IPTG-inducible *opa_52_* gene were a kind gift from Dr. Scott Gray-Owen (University of Toronto, Canada). Frozen stocks were streaked on a LB plate containing 100 µg Ampicillin, and incubated at 37°C overnight. After 24 h, a single colony was picked and streaked on a LB/Ampicillin plate containing 0.1 mM IPTG, and incubated at 37°C overnight.

FITC was prepared at a final concentration of 1 mg/ml in 50 mM carbonate, 150 mM NaCl buffer (pH 9.2). Bacteria were collected in 1 ml PBS/Mg/Ca and re-suspended by gently pipetting and pelleted at 3000 rpm for 3 min. After removal of the supernatant, the bacteria were re-suspended in 500 µl of FITC solution and incubated at room temperature with gentle shaking in the dark for 30 min. After washing (5×) the bacteria were re-suspended in PBS/Mg/Ca with 1% BSA to quench remaining FITC. The labeled bacteria were diluted to a working ratio of 30 bacteria to 1 neutrophil.

### Pronuclear microinjection of fertilized mouse oocytes

All experiments involving live animals were conducted with the approval of the City of Hope institutional animal care and use committee. Mice used in the derivation of transgenic mice were maintained on a 14 hr light/10 hr dark cycle. Female mice (FVB/NJ, Jackson Laboratories) were superovulated with 5 IU of pregnant-mare serum-gonadotropin (National Hormone and Peptide Program, UCLA, Los Angeles, CA) followed 48 hours later by 5 IU of human-chorionic gonadotropin (Sigma-Aldrich, St. Louis MO) and mating to fertile FVB/NJ males. Fertilized oocytes were collected ∼12 to 14 hours after the middle of the dark cycle, and treated briefly with bovine hyaluronidase in M2 medium to remove the cumulus cells. Immediately prior to microinjection the purified BAC DNA was diluted to a concentration of 3 ng/µL in 1 mM Tris-HCl (pH 8.0), 0.1 mM EDTA, 100 mM NaCl containing 30 mM spermine/70 mM spermidine [31]. The BAC DNA solution was microinjected into the pronuclei of fertilized oocytes using standard techniques, and the manipulated oocytes were transferred surgically into the oviducts of 0.5 dpc pseudo-pregnant recipient female mice and allowed to develop to full term.

### PCR analysis to screen positive pups

The initial screening of founder mice for the human *CEACAM1* transgene was carried out by PCR analysis on mouse tail DNA. Briefly, tail samples (1–3 mm) were digested in 0.25 mL 0.4 N NaOH at 95°C for 10 to 15 min until tails were fully digested. Followed by adding 0.375 mL neutralization buffer (0.5 M Tris pH 8.0+20 mM EDTA). Digests were then diluted 10-fold. PCR were then performed by using Bio-Rad iCycler PCR (Bio-Rad Laboratory, Hercules, CA) with 2X fideliTaq PCR master mix (USB, Cleveland, Ohio). Two µL of diluted tail DNA was added into a final 20 µL volume reaction. The sense strand primer 5′-CACCATGCCCAGCTAATTTT-3′ and antisense strand primer 5′-GTGGTCTGTTGCTCCCAGAT-3′ only target the human *CEACAM1* gene. The amplified product is 260 bp. The amplification parameters were initiated at 95°C for 5 min, then 95° C for 30 s, 53°C for 30 s, and 72°C for 30 s for 35 cycles, followed by 7 min at 72°C for the final extension. The primer sets for the mouse *CEACAM1* gene are: sense 5′-ATGAACAGTGAGGCAGTCCC-3′ and antisense 5′-CAGAGGAAGAGTGTCCAGGC-3′. The amplification parameters were initiated at 95°C for 5 min, then 95° C for 30 s, 55°C for 30 s, and 72°C for 30 s for 35 cycles, followed by 7 min at 72°C for the final extension. The final product length is 155 bp.

### Western blot analysis

Eight weeks old F2 pups (one human *CEACAM1* gene positive and one negative by PCR) were euthanized by CO_2_ inhalation. Liver, heart, lung, brain, kidney, spleen, small intestine (ileum), and neutrophil (from peripheral blood) or fecal pellets were homogenized and lysed in 1% NP-40 lysis buffer. Cell lysates were incubated on ice for 30 min, followed by centrifugation for 30 min at 4°C at 14000 rpm. Total protein (50 µg) was separated by sodium dodecyl sulfate-gel polyacrylamide electrophoresis, transferred to nitrocellulose membranes and probed with either anti-murine CEACAM1 mAb CC1 (1 µg/mL, kind gift from Dr. Kathryn Holmes, University of Colorado health Sciences, Denver, CO) and anti-β-actin antibody (1 µg/mL, Abcam, Cambridge, UK) antibody or anti-human CEACAM1 mAb T84.1 (1 µg/mL) [32]. Signals were detected on the Odyssey Infrared Imaging System (LI-COR Biosciences, Lincoln, NE, USA).

### Immunohistochemistry

Mice were euthanized by CO_2_ inhalation. Tissues were collected and fixed in 4% paraformaldehyde overnight. Immunohistochemical staining was performed on 5 µm thick sections prepared from formalin fixed, paraffin-embedded blocks. Sections were deparaffinized in xylene and rehydrated in a graded alcohol series. Slides were quenched in 3% hydrogen peroxide, steamed for antigen retrieval in DIVA/citrate buffer (pH 6.0) (BIOCARE ref; DV2004MX) for 20 minutes, and incubated in Rodent block (BIOCARE MM HRP- Polymer Kit ref: MM510G) for 30 minutes. The slides were incubated overnight at 4°C with either CC1 1∶250 (8 µg/mL) or T84.1 mAbs at a 1∶250 dilution (15 µg/mL). The following day, slides were washed, incubated in Mouse Polymer-HRP (BIOCARE MM HRP- KIT ref: MM510G) for 20 minutes, and treated with the chromogen diaminobenzidine tetrahydrochloride (DAB), counterstained with hematoxylin, dehydrated through grades of alcohols and xylenes and mounted with permanent mounting media. Blood smears on positive charged glass slides (Surgipath, Richmond, IL), fixed in methanol for 15 sec and stained as above. Mouse anti-human IgG1 (eBioscience, San Diego, CA) was used as an isotype control.

### Preparation of bone marrow cells and spleen cells and isolation of neutrophils

Transgenic and wild type FVB mice, born within the same litter, were euthanized by CO_2_ inhalation. The bone marrow cells were flushed from femurs and tibias by using a 23-gauge needle with 10 mL of cold PBS. Single cell suspensions were prepared by flushing the cell solution twice through a 22-gauge needle and passing the cell solution through a sterile 40-µm nylon mesh. Spleens were removed aseptically, injected with 2 mg/mL of collagenase D and 20 µg/mL of DNase I (Roche Diagnostics, Mannheim, Germany) in Hanks solution, minced, incubated for 30 min at 37°C under 5% CO_2_, and teased through a 40 µm nylon mesh. Red blood cells in bone marrow cells and spleen cells were lysed twice using Red Blood Cell Lysing Buffer Hybri-Max (Sigma-Aldrich, Saint Louis, MO 63103). Cells were washed twice with RPMI-1640 medium and subsequently suspended in RPMI-1640 culture medium containing 10% FBS and antibiotics. Cell viability was determined by trypan blue dye exclusion.

Mouse Gr-1^+^ granulocytes from bone marrow were positively isolated using Anti-Ly-6G MicroBead Kit according to the manufacturer's protocol (Miltenyi Biotec, Bergisch Gladbach, Germany). Human neutrophils were isolated from discarded blood. This study was approved by the Institutional Human Subject's Review Board (City of Hope National Medical Center). Neutrophils were isolated by centrifugation over Ficoll-Paque Plus (GE Healthcare Biosciences, Pittsburgh, PA, USA) density gradient. Cell purities were determined by forward light-scatter/side light-scatter gating of cells stained with PerCP-Cy5.5-conjugated anti-CD16 mAb using a flow cytometer (FACSCanto™ II, BD Biosciences) and neutrophil purity was more than 95% [33].

### Neutrophil binding assay

Neutrophils from mice or human were suspended at 1×10^6^ cells/mL in RPMI-1640 medium. Degranulation was induced by adding fMLF (Sigma-Aldrich, Saint Louis, MO 63103) to a final concentration of 100 nM followed by incubation at 37°C for 30 min. FITC-labeled *E. coli* (30×10^6^ cells/mL) with either Opa_52_ or vector control were added to neutrophils (neutrophil/bacteria; 1/30) and incubated at 37°C for 60 min. Infection was arrested by washing the neutrophils with PBS three times.

### Confocal microscopy

Neutrophils were fixed in 4% paraformaldehyde with 50 mM HEPES buffer for 20 min at room temperature, followed by 15 min of 0.1% triton X-100, and 15 min of 0.2% BSA in PBS. Anti-human CEACAM1 mAb T84.1 was then added to the fixed cells at a final concentration of 1 µg/mL for 1 hour. After 3 washes with PBS, goat anti-mouse Alexa 555-conjugated antibody (Invitrogen, Eugene, OR) was added at the concentration of 2 µg/mL for another 30 min followed by 3 washes with PBS. Stained cells were plated on glass slides with Hard Set mounting medium and counterstained with DAPI from Vector Laboratories Inc (Burlingame, CA 94010, USA). Confocal microscopy was performed on a Zeiss Model 310 (Oberkochen, Germany) confocal microscope.

### Flow cytometry analyses

Single cell suspensions from bone marrow or spleen from transgenic or wild-type mice were blocked by anti-mouse CD16/32 antibody (Clone: 93, Biolegend, San Diego, CA 92121), and stained with either the anti-murine CEACAM1-PE (clone CC1, Biolegend, San Diego, CA 92121) or anti-human CEACAM1-PE (Clone: 283340, R&D Systems Inc, Minneapolis, MN 55413). The bone marrow cells were counter stained with anti-Gr-1-APC (Clone: RB6-8C5) and anti-CD11b-APC-Cy7 (Clone: M1/70, BD Biosciences, San Jose, CA 95131) to identify neutrophils, and the spleen cells were counter stained with anti-CD3-FITC (Clone: 17A2) and anti-B220-APC (Clone: RA3-6B2, BD Biosciences, San Jose, CA 95131) to identify B and T cells. The cell surface expression of murine CEACAM1 and human CEACAM1 was analyzed on FACSCanto™ II (Becton Dickinson) with Flowjo software (Becton Dickinson).

Single cell suspensions (1×10^6^/mL) from spleen from transgenic or wild-type mice were cultured in a 24-well plate for 72 hours in the absence or presence of 1 µg/ml plate-bound anti-CD3 mAb (145-2C11) and 2 µg/ml soluble anti-CD28 (37.51; BD Pharmingen). Cells were harvested, stained and analyzed as above methods by FACS.

### Reverse transcriptase PCR

Total RNA was isolated from transgenic mouse liver, kidney and small intestine by using Trizol® method (GIBCO-BRL/Invitrogen Corp., Carlsbad, CA). Briefly, samples were homogenized in 800 ìL Trizol® (GIBCO-BRL/Invitrogen Corp., Carlsbad, CA), followed by the addition of 160 ìL chloroform. After centrifugation at 4°C at 14,000 rpm for 15 min, the top layer was transferred to a clean microcentrifuge tube and 400 ìL of 2-propanol was added, incubated at room temperature for 20 min and centrifuged at 14,000 rpm for 10 min at 4°C. Total RNA was washed in 75% ice-cold ethanol once and after 5 min was centrifuged at 14,000 rpm at 4°C. RNA pellets were air dried and dissolved in 50 ìL RNase-free water. Complementary DNA (cDNA) was prepared using the Omniscript® reverse transcription system (Qiagen, Inc., Valencia, CA) according to the manufacturer's protocol. The reverse transcription system contains oligo primers, RNase inhibitor, MMLV reverse transcriptase, dNTP mix, buffer and 3 ìg total RNA in a final volume of 20 ìL. The reaction mixture was incubated at 42°C for 1 h. CEACAM1 isoforms were detected by PCR on a Bio-Rad iCycler PCR (Bio-Rad Laboratory, Hercules, CA) with 2X fideliTaq PCR master mix (USB, Cleveland, Ohio). cDNA (5 µl) was added to a final 25 µL volume reaction. The sense strand primer 5′-AACGTCACCCAGAATGAC-3′ and antisense strand primer 5′-CTCAGGACCACTCCAATGA-3′ target human *CEACAM1* gene. The amplified products are 1118 bp (4L), 1066 bp (4S), 831 bp (3L), 779 bp (3S). The amplification parameters were initiated at 95°C for 5 min, then 95° C for 30 s, 55°C for 90 s, and 72°C for 30 s for 38 cycles, followed by 7 min at 72°C for the final extension. For mouse CEACAM1, the sense strand primer 5′-GACTCTGTCGACGACATGGAGCTGGCCTCAGCACATC-3′ and antisense strand primer 5′-GACTCTATCGATTCACTTCTTTTTTACTTCTGAATAAAC-3′ were used to PCR amplify the murine *Ceacam1* cDNAs. The four RT-PCR amplified products for murine Ceacam1 correspond to the 4L, 4S, 2L and 2S mRNA splice isoforms. The amplification parameters were same as above.

## Results

### Generation of human CEACAM1 transgenic mice

**Figure 1:**
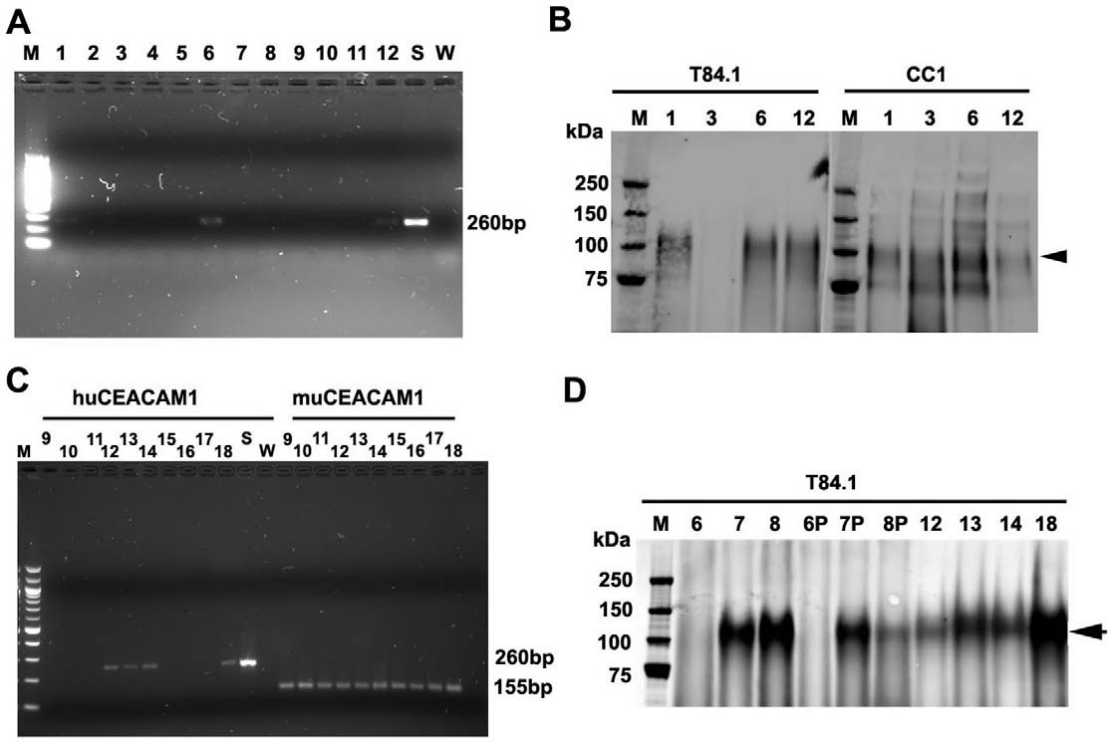
Transgenic mouse screening. **A**. PCR screening for *CEACAM1* expression. Pups 1 to 12 were born after pronuclear microinjection of BAC2. Founders 1, 6 and 12 were positive using *CEACAM1* primers. **B**. Western blots of feces using anti-human CEACAM1 mAb demonstrated that BAC2 founders 1, 6 and 12 expressed human CEACAM1 in their gastrointestinal tracts. **C**. PCR analysis of the F1 generation from founder 12. About 50% of the pups were positive (left side) for human *CEACAM1* and 100% positive for murine CEACAM1 (right side). **D**. Western blot analysis of feces using anti-human CEACAM1 mAb. Pups 7, 8, 12, 13, 14, and 18 from founder BAC2-12 were positive. Most fecal samples were collected in the morning (no label) or in the evening (7P, 8P) to determine if time of collection affected the analysis.

Three BAC clones containing the human *CEACAM1* gene were microinjected into the pronuclei of fertilized oocytes of FVB N/J mice and founder transgenics were generated with BAC2 and BAC5 clones. BAC2Clone (214 kb) covered a large region proximal to human *CEACAM1* on chromosome 19, and two other BAC clones (BAC3 and BAC5; 138, and 211 kb, respectively) covered shorter regions flanking the human *CEACAM1* gene. Although litters were obtained from mice fertilized with all three microinjections, only those from BAC2 and BAC5 transmitted the germline gene to their offspring (**Fig. 1A** **and data not shown**), and only those from BAC2 exhibited expression of the huCEACAM1 protein in their feces (**Fig. 1B**). Since CEACAM1 is heavily expressed in the GI tract, from the liver throughout the small and large intestine, and is shed into the lumen of the gut like CEA [34], we used its detection in the feces as an early diagnostic test for protein expression. As can be seen in **Fig. 1B**, both murine and human CEACAM1 were detected in the feces of the founders. Only the offspring from founder 12 (BAC2) expressed the transgene. As expected, roughly 50% of the pups were positive for human *CEACAM1* by both PCR analysis of tail samples (**Fig. 1C**) and western blot analysis of fecal samples (**Fig. 1D**) of the F1 generation from founder 12 (BAC2). Further breeding of the BAC2-12 transgenic mice demonstrated transmission of an expressed CEACAM1 transgene from the F1 to F2 generation at the expected (∼0.5) frequency for heterozygotes. No attempt was made to breed the BAC2-12 line to homozygosity at this stage of the study. Expression of the human CEACAM1 transgene was not detected in any of the BAC5 founders or their F1 offspring. Given the small number of litters obtained from each microinjection, it was not feasible for us to evaluate the success rate in terms of stable integration and expression of the various BAC clones.

**Figure 2:**
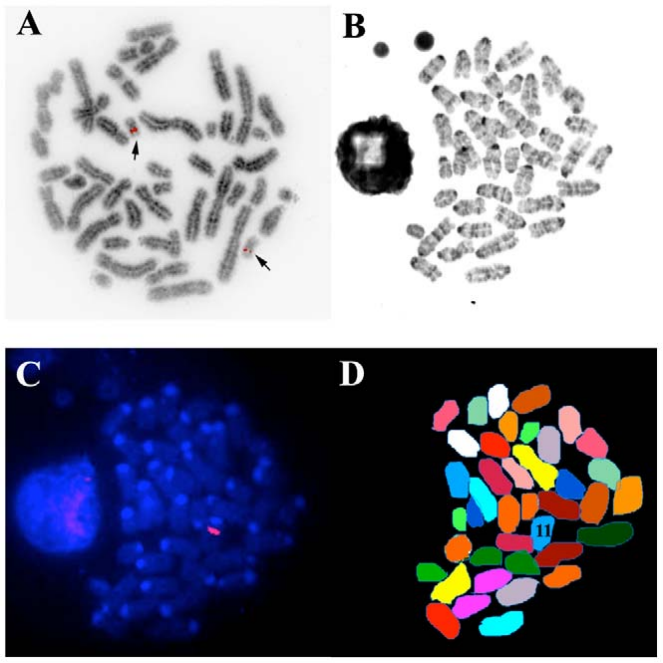
Chromosomal location of human *CEACAM1* gene in transgenic mice. **A**. Human chromosomal spread showing hybridization of human *CEACAM1* gene probe to chromosome 19 (arrows). **B**. Murine chromosomal spread of the transgenic mice. **C**. Hybridization of human *CEACAM1* gene probe to the murine chromosomal spread, counterstained with DAPI. **D**. Painting of the murine chromosomal spread from the transgenic mouse. Chromosome 11 is labeled, demonstrating a single integration site on one chromosome (heterozygous).

**Figure 3:**
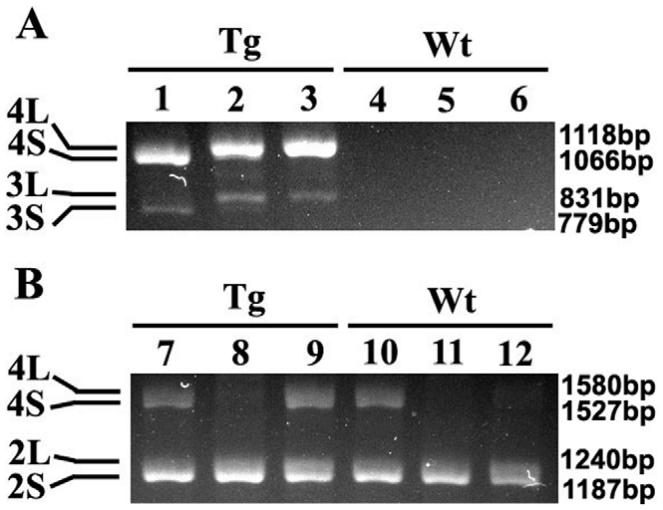
*CEACAM1* mRNA isoform expression in BAC2-12 transgenic mouse liver, kidney and small intestine. **A.** RT-PCR analysis for *CEACAM1* isoforms in transgenic (1–3) or wild type mice (4–6) in liver (1, 4), kidney (2, 5), and small intestine (3, 6). **B.** RT-PCR analysis for *Ceacam1* isoforms in transgenic (7–9) or wild type mice (10–12) in liver (7,10), kidney (8,11), and small intestine (9,12).

The chromosomal insertion point for the BAC clone is random in transgenic animals produced by pronuclear microinjection. Using human specific probes for CEACAM1, we were able to demonstrate that the human gene is on chromosome 19 of human cells (**Fig. 2A**), as expected, and the BAC2 clone containing the human *CEACAM1* gene was on chromosome 11 of the transgenic mice (**Fig. 2C–D**). Although we did not confirm the location of the endogenous murine *Ceacam1* gene, it has been reported to be on chromosome 7 [35], [36]. Thus, the murine *Ceacam1* and human *CEACAM1* genes are on different chromosomes of the transgenic mice. Since mRNA from the human *CEACAM1* can be alternatively spliced, it was also important to determine if the expected splice forms were present. For this analysis, RNA was isolated from BAC2-12 transgenic mouse liver, kidney and small intestine and analyzed by PCR using specific primers for the major splice forms expected in human CEACAM1. As seen in **Fig. 3A**, transgenic mouse liver expressed human CEACAM1-4S and -3S isoforms, while transgenic kidney and small intestine expressed the -4L and -3L isoforms. Analysis of the endogenous expression of murine CEACAM1 isoforms revealed the -4S and -2S isoforms in the liver and intestine, while the kidney expressed mainly the -2S isoform (**Fig. 3B**). Since mice express a highly homologous second allele, namely CEACAM2, mainly expressed in the testes and kidney, we cannot be sure if the kidney PCR products are from murine CEACAM1 or CEACAM2. The differences in tissue specific CEACAM1 isoform expression between mouse and man has been previously described [37], [38].

### Tissue expression patterns of huCEACAM1 in transgenic animals

**Figure 4:**
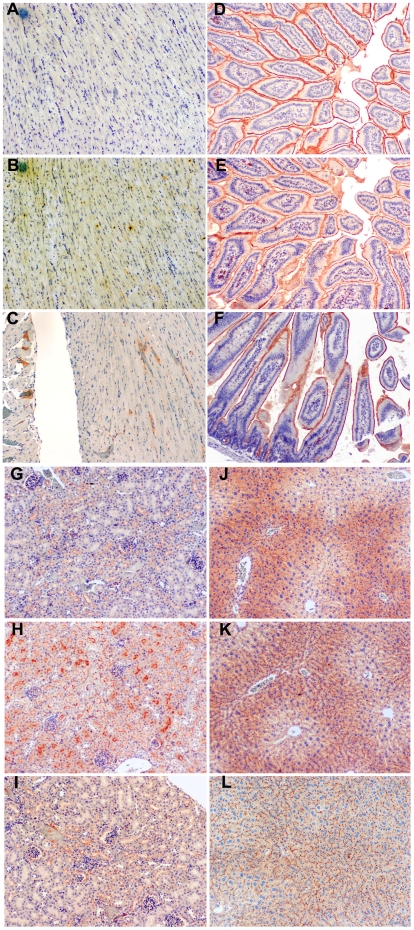
Immunohistochemistry staining for human and murine CEACAM1 in BAC2-12 transgenic and wild type mice. Heart (**A–C**), small intestine (**D–F**), kidney (**G–I**), and liver (**J–L**) in transgenic (**A, B, D, E, G, H, J, K**) and wild type mice (**C, F, I, L**) by anti-murine CEACAM1 mAb (CC1) (**A, D, G, J, C, F, I, L**) or anti-human CEACAM1 mAb (T84.1) (**B, E, H, K**). Mag. 50X.

Having obtained successful germline transmission of the human *CEACAM1* gene and CEACAM1 protein expression in the BAC2-12 line of mice, we analyzed tissues from the transgenic mice by immunohistochemical staining to determine if expression of the human and murine genes was similar. First we analyzed heart as a negative tissue to eliminate the possibility that inappropriate expression occurred. Neither murine nor human CEACAM1 was expressed in the hearts of BAC2-12 transgenic mice, and wild type FVB mouse heart is shown as an additional control (**Fig. 4A–C**). Three tissues that exhibit high expression of CEACAM1 in both mouse and human are the intestine, kidney, and liver. As seen in **Fig. 4D–F**, the expression of human CEACAM1 is similar to murine CEACAM1 in the small intestine in terms of distribution and intensity. As expected, both the murine and human CEACAM1 are expressed on on the luminal surface of intestinal epithelial cells. In the case of the kidney, the expression of human CEACAM1 is noticeably higher than the expression of murine CEACAM1 (compare **Fig. 4H** to **Fig. 4G**, transgenic, and **Fig. 4I**, wild type FVB). This difference may be explained by the fact that mice have two *Ceacam* genes (*Ceacam1* and *Ceacam2*) that are differentially expressed in the kidney [35], [37], while humans have a single CEACAM1 gene. Thus, the expression of the human gene in the kidney may reflect the combined expression of the two genes in mouse; however, the anti-murine CEACAM1 antibody used here does not detect murine CEACAM2. The reason for the differential expression of the two mouse genes in the kidney is unexplained, but may reflect a role for the kidney in insulin clearance (see below). In the case of the liver, the staining pattern for murine and human CEACAM1 in the transgenic animals is identical (**Fig. 4J–K**). The pattern shows highest expression around the portal triads with decreased expression around the portal artery. This may reflect the known function of CEACAM1 in effecting insulin clearance [39]. The observed CEACAM1 expression pattern follows the entrance of blood from the portal circulation and exit via the portal artery. The staining pattern for the murine CEACAM1 of wild type mice liver (**Fig. 4L**) is similar but less intense. Further quantitative studies are required to determine if there is a real difference in degree of expression of the endogenous murine *Ceacam1* gene between the wild type and transgenic animals.

**Figure 5:**
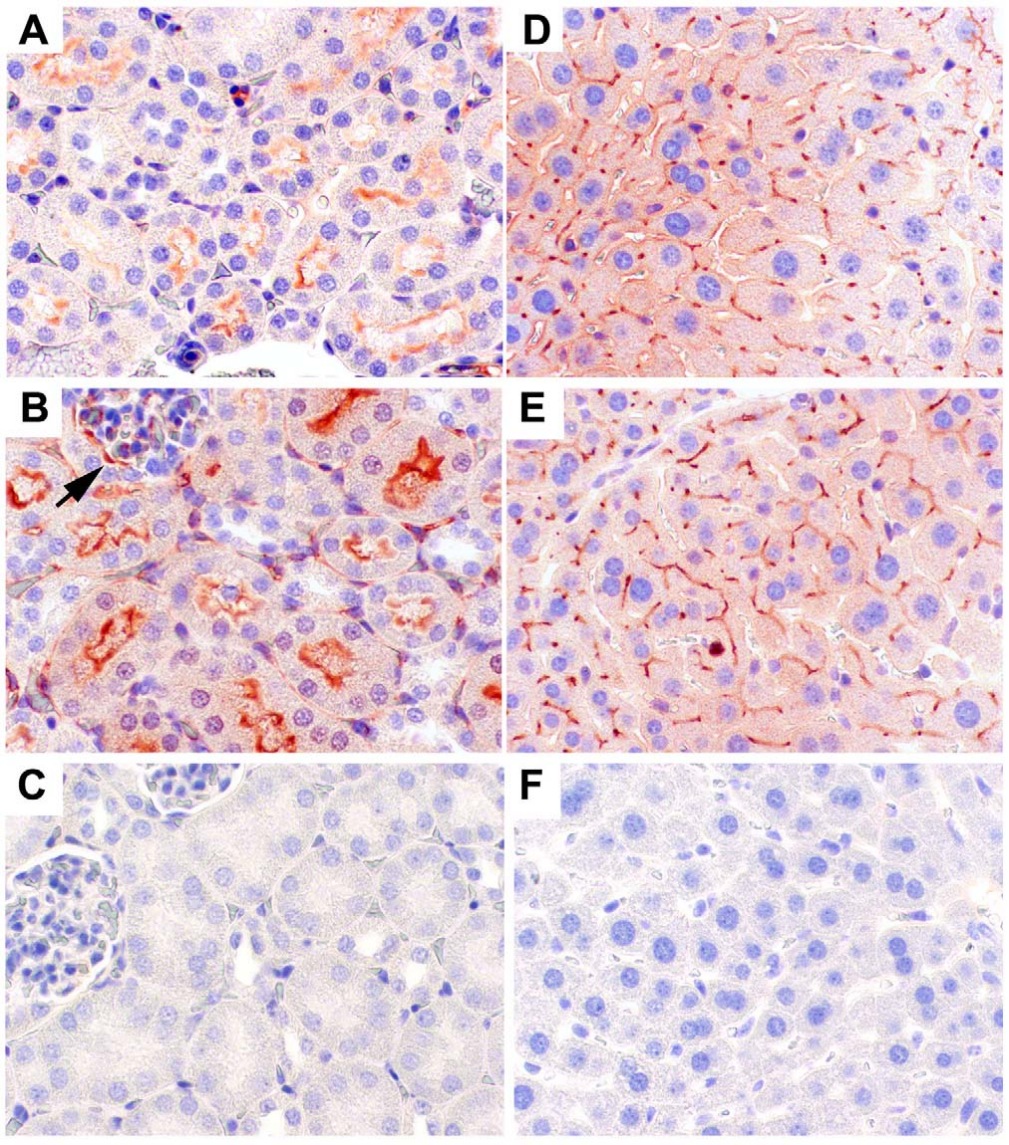
Immunohistochemistry staining for human and murine CEACAM1 in kidney and liver of BAC2-12 transgenic and wild type mice. **A**. Wild type mouse kidney. **B**. Transgenic mouse kidney. **C**. Transgenic mouse kidney with isotype control antibody. **D**. Wild type mouse liver. **E**. Transgenic mouse liver. **F**. Transgenic mouse liver with isotype control antibody. Note that human CEACAM1 expression is higher than murine CEACAM1 in transgenic mouse kidney. However, in the liver, murine and human CEACAM1 expression in transgenic mice was similar. Bowman's capsule in the kidney is indicated by an arrow (**B**). Mag. 200X.

The high expression of CEACAM1 in kidney and liver was examined at higher magnification. As seen in **Fig. 5A–B**, the expression pattern for human CEACAM1 is identical to murine CEACAM1, but more intense. The pattern is consistent with the highest levels of expression in proximal tubules (less open lumen) and the lowest in distal tubules (more open lumen). In all cases the staining is on the luminal surface of the renal tubules. CEACAM1 is also expressed on the luminal surface of epithelial cells of the Bowman's capsules surrounding the glomeruli in the kidney (arrow, **Fig. 5B**). In the case of the liver, the expression pattern of both murine and human CEACAM1 in the transgenic animals is confined to the bile canaluculi (**Fig. 5D–E**). This expression pattern has been described previously [40] and suggests the possibility that the mechanism of insulin clearance by CEACAM1 [39] may be related to the function of bile canaliculi.

**Figure 6:**
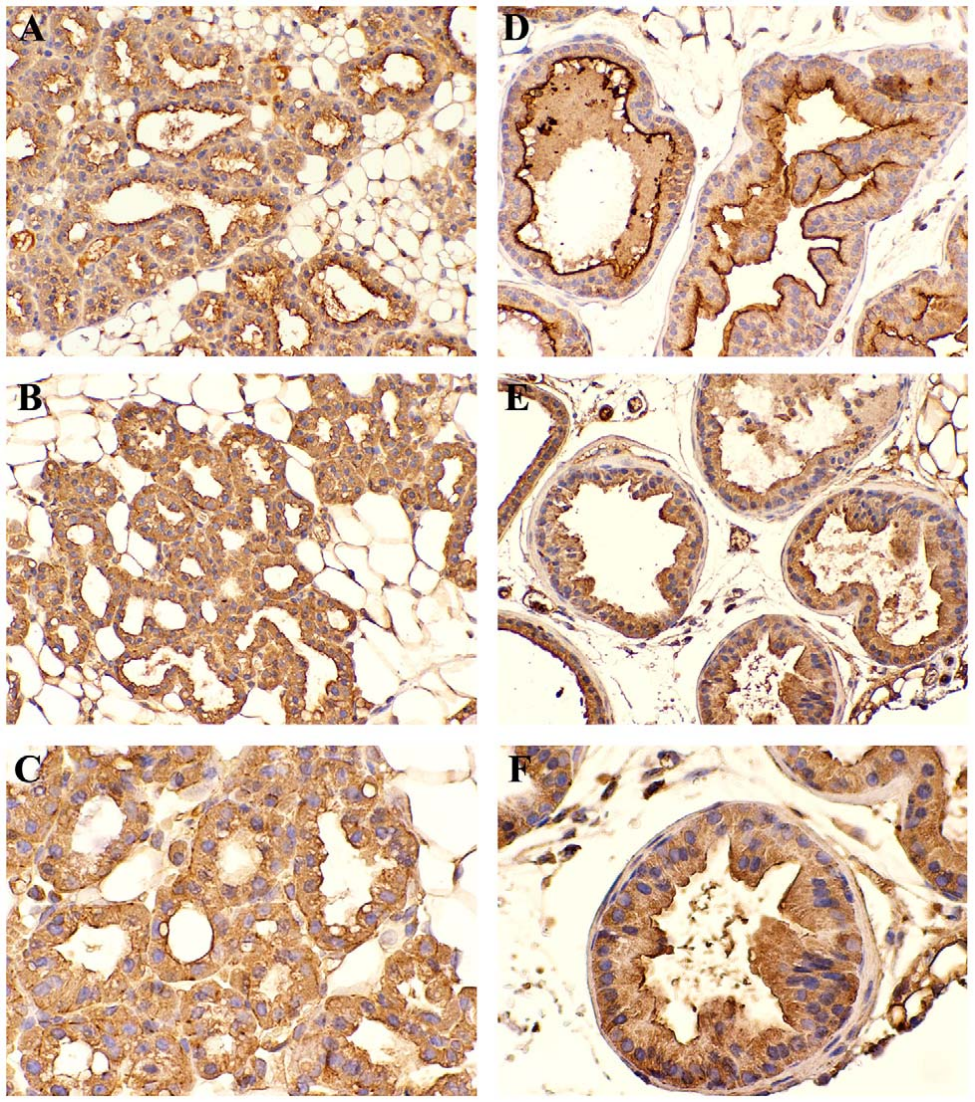
Immunohistochemistry staining for murine and human CEACAM1 in the mammary and prostate glands of BAC2-12 transgenic mice. **A**. Murine CEACAM1 expression in the mammary gland (Mag 50X). **B** and **C**. Human CEACAM1 expression in the mammary gland (Mag 50X and 200X, respectively). **D**. Murine CEACAM1 expression in the prostate gland (Mag 50X). **E** and **F**. Human CEACAM1 expression in the prostate gland (Mag 50X and 200X, respectively).

The consistent luminal expression of CEACAM1 on epithelial cells supports the hypothesis that CEACAM1 plays a role in lumen formation in several tissues [5], [6], [7]. This role has been documented in mammary epithelial cells grown in either a 3D culture [5], [6], [7] or in humanized mammary fat pads in NOD/SCID mice [41], [42]. CEACAM1 is also expressed on the luminal surface of prostate glands [43], and like its expression in the mammary gland, is down-regulated in cancer [44]. As a first step in using these transgenic animals as a model for mammary and prostate cancer we examined its expression in these tissues. In the case of the mammary gland, it was necessary to study pregnant females, since the mammary gland is minimally developed in non-pregnant females. As shown in **Fig. 6A–C**, there was strong luminal expression of both murine and human CEACAM1 in the mammary glands of pregnant transgenic mice. There was also evidence for cytoplasmic expression, perhaps indicating active transport of CEACAM1 from the site of synthesis to the lumen. Similar results were seen for the prostate glands of male human *CEACAM1* transgenic mice (**Fig. 6** **D–F**). These results suggest that these mice may be an appropriate model for studying the role of human CEACAM1 in tumorigenesis within these organs.

### Binding of *E. coli*-Opa_52_ by neutrophils

**Figure 7:**
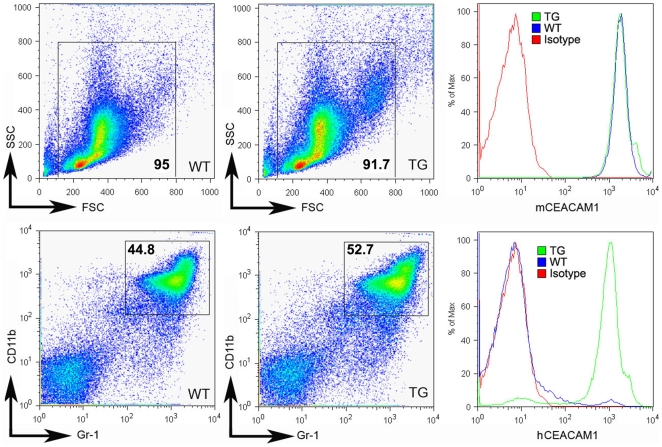
Expression levels of murine and human CEACAM1 on bone marrow neutrophils of BAC2-12 transgenic and wild type mice. Single cell suspensions of bone marrow from TG or WT mice were stained with either anti-murine CEACAM1-PE or anti-human CEACAM1-PE and co-stained with anti-Gr-1-APC or anti-CD11b-APC-Cy7. Histograms on the right are from gated cells as shown. Results shown are representative of three independent experiments with similar results.

**Figure 8:**
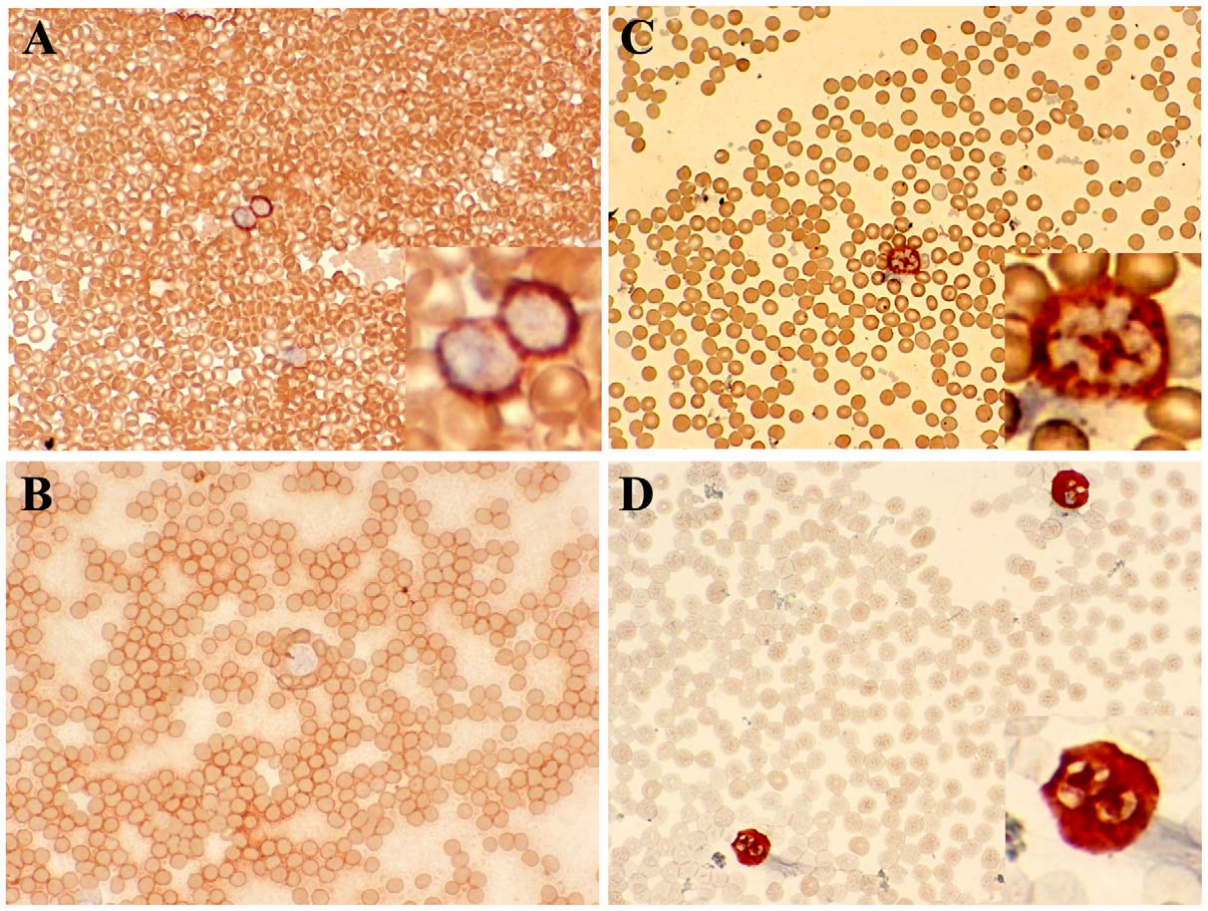
Immunohistochemistry staining of blood neutrophils from BAC2-12 transgenic and wild type mice. **A**. Transgenic mouse blood neutrophils stained with anti-human CEACAM1 antibody. Mag. 200X (inset photo enlarged 4X). **B**. Wild type mouse blood neutrophils stained with anti-human CEACAM1. Mag. 200X. **C**. Transgenic mice blood neutrophils stained with anti-mouse CEACAM1. Mag. 200X (inset photo enlarged 4X). **D**. Human neutrophils stained with anti-human CEACAM1. Mag. 200X (inset photo enlarged 4X).

**Figure 9:**
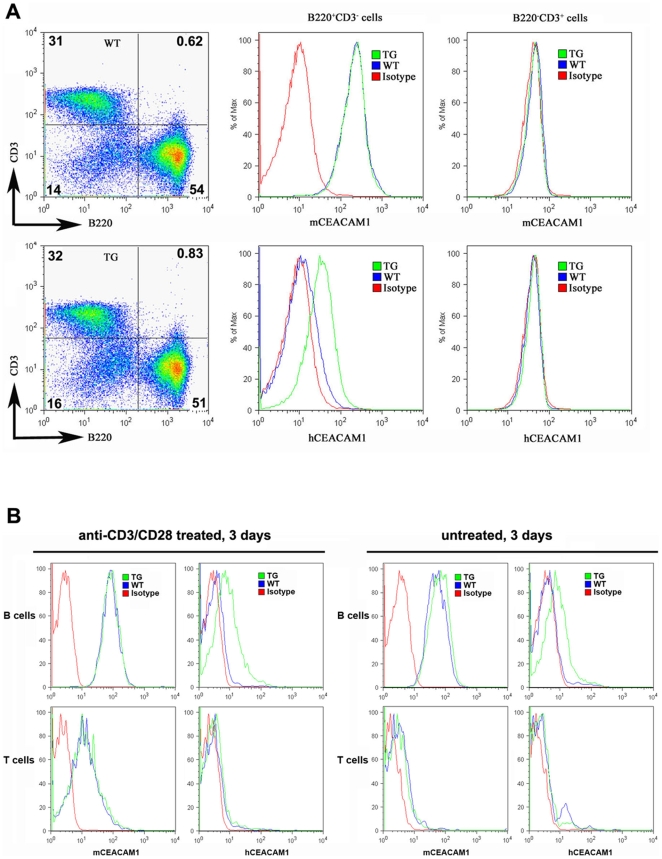
Expression levels of murine and human CEACAM1 on spleen B and T cells of BAC2-12 transgenic and wild type mice. Freshly isolated spleen cells (**A**) and Spleen cells treated with 1 µg/mL anti-CD3 and 2 µg/mL anti-CD28 mAb for three days (**B**) from TG or WT mice were stained with either the anti-murine CEACAM1-PE or anti-human CEACAM1-PE and co-stained with anti-CD3-FITC and anti-B220-APC. Results shown are representative of three independent experiments with similar results.

Another potential use of human *CEACAM1* transgenic mice is to study pathogenesis where CEACAM1 functions as the pathogen receptor. The situation is especially critical in the case of *Neisseria gonorrhoeae* infections where there is a pressing need for the development of a vaccine [2]. Since the high expression of CEACAM1 on neutrophils plays an important role in its pathogenesis, we determined the expression levels of huCEACAM1 on neutrophils isolated from the bone marrow and blood of the transgenic animals. As shown in **Fig. 7**, the expression of murine and human CEACAM1 in the transgenic animals was strong. As shown in **Fig. 8(A–C)**, the staining of murine and human CEACAM1 was strong for neutrophils in blood smears taken from the human *CEACAM1+/−* transgenic animals, while staining for human CEACAM1 was negative for a *CEACAM1−/−* littermate. For purposes of comparison, neutrophils from a human blood smear were stained (**Fig. 8D**). Notably, human neutrophils stain more intensely than those from the transgenic animals, suggesting a possible effect of an expression difference between the species. In addition, we examined the expression of human CEACAM1 in the spleen of transgenic animals. Based on cell surface staining, there was no expression on T-cells and significant expression on B-cells (**Fig. 9A**). Consistent with these results, CEACAM1 has been shown to be inducible on both murine [45] and human T-cells [14]. The expression of CEACAM1 on murine [46] and human [17], [47] B-cells has been previously reported. Activation of splenocytes from transgenic animals by anti-CD3/28 antibodies for three days revealed increased expression of murine, but not human CEACAM1 in T-cells (**Fig. 9B**). B-cell expression of murine and human CEACAM1 is shown for comparison.

**Figure 10:**
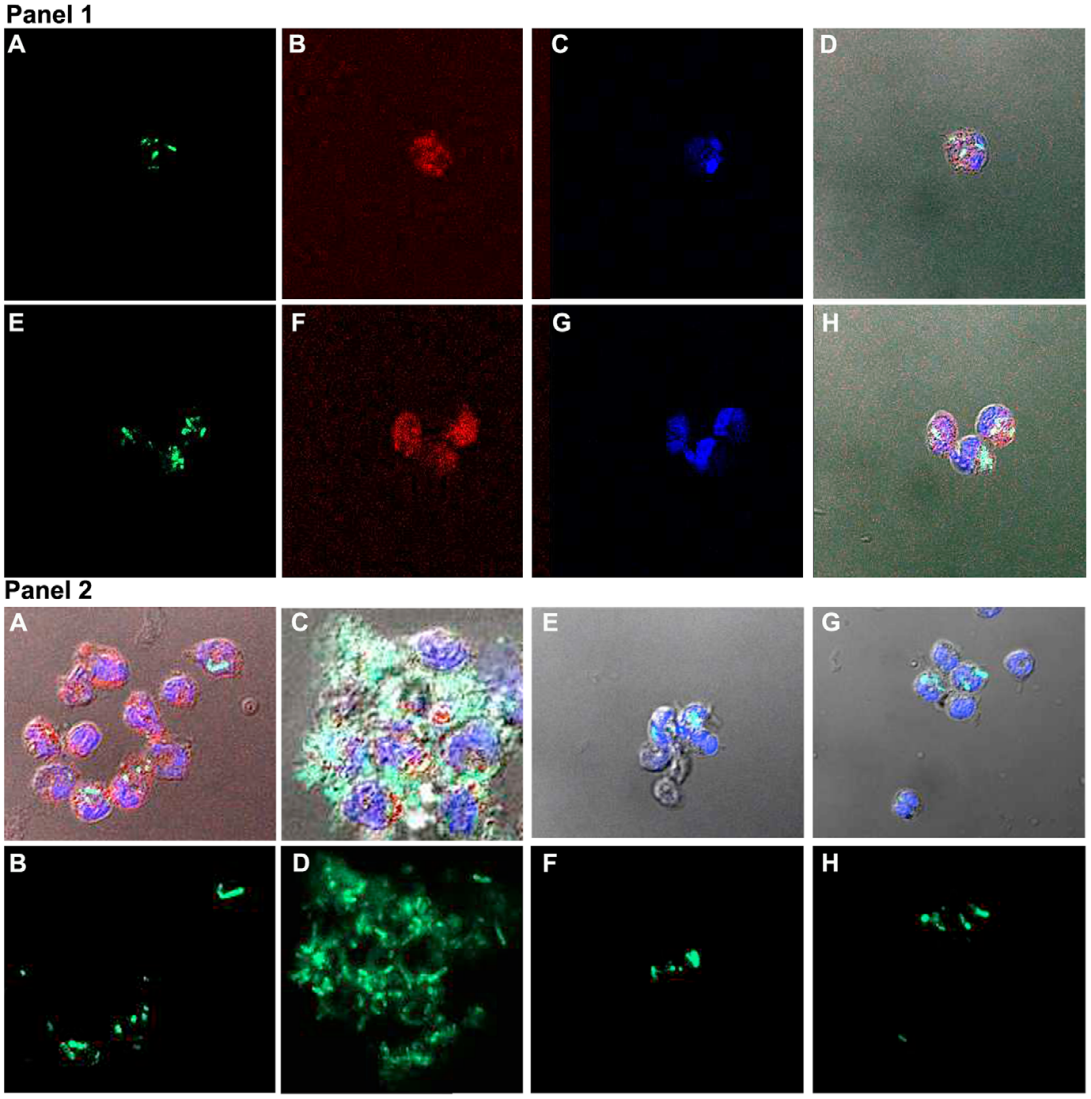
Confocal analysis of transgenic mouse neutrophils undergoing binding of *E. coli* expressing recombinant Opa_52_ protein. **Panel 1**. *E. coli* vector control or Opa_52_ phagocytosed by human neutrophils. **A**. Vector control *E. coli* (FITC labeled, green). **B**. Same as A, stained with anti-human CEACAM1 mAb Alexa 555 labeled (red). **C**. Same as A, stained with DAPI (blue). **D**. Overlay of A–C on phase contrast image. Mag 300X. **E**. Opa_52_ *E. coli* (FITC labeled, green). **F**. Same as E, stained with anti-human CEACAM1 Mab Alexa 555 labeled (red). **G**. Same as E, stained with DAPI (blue). **H**. Overlay of E–G on phase contrast image. Mag 300X. **Panel 2**. *E. coli* vector control or Opa_52_ phagocytosed by neutrophils from transgenic (A–D) or wild type (E–H) mice. **A, E.** Vector control *E. coli* (overlay with FITC, Alexa 555, and DAPI on phase contrast image). **B, F.** vector control *E. coli* (FITC labeled, green). **C, G.** Opa_52_ *E. coli*. (overlay with FITC, Alexa 555, and DAPI on phase contrast image) **D, H.** Opa_52_ expressing *E. coli* (FITC staining).

Having demonstrated expression of human CEACAM1 on the neutrophils of the transgenic animals, it was important to demonstrate that they could bind Opa proteins, the requisite *Neisseria gonorrhoeae* ligand for human CEACAM1 [8]. For this analysis, we utilized *E. coli* expressing recombinant Opa_52_ protein. As shown in **Fig. 10**, primed neutrophils from transgenic animals were able to bind *E.coli-*Opa_52_ much greater than *E. coli*-vector control. Controls included binding of the parental (Opa-) strain on wild type neutrophils and Opa52-expressing bacteria binding to human neutrophils. These results suggest that the neutrophils in the transgenic animals may mimic the behavior of human neutrophils towards binding of *Neisseria* pathogens.

## Discussion

The generation of human CEACAM1 transgenic mice is an important model to demonstrate equivalence in function of the murine and human gene orthologs. Since pathogens that utilize human CEACAM1 as their receptor do not recognize murine CEACAM1, such a model is essential to study their pathogenesis. Furthermore, in order to demonstrate that CEACAM1 specific anti-pathogen vaccines are effective, the model must mimic the human tissue specific pattern of CEACAM1 expression. Thus, our approach was to generate transgenic mice that expressed the entire human *CEACAM1* gene rather than a cDNA for CEACAM1. Although we attempted to generate several lines using BAC clones that spanned different regions of chromosome 19 encoding the *CEACAM1* gene, only a single line that actually expressed the protein was generated. Given the large number of variables that affect both integration, expression, and transmission of a gene, the reason for this result is unknown. Fortunately, the BAC2-12 line obtained transmitted the transgene from the founder mouse to F1 and F2 generations and demonstrated the expected frequency for heterozygotic expression in subsequent generations. Furthermore, human specific splice forms were found in three major tissues (liver, intestine and kidney) of known CEACAM1 expression and tissue staining for human CEACAM1 demonstrated the expected staining pattern, namely at the lumen of epithelial cells.

Both murine *Ceacam1* and human *CEACAM1* genes produce multiple mRNA splice isoforms. Murine *Ceacam1* generates mRNAs having mainly 4 or 2 ectodomains with long or short cytoplasmic domains. Indeed, the expected major splice isoforms of murine *Ceacam1* were found in the liver, intestine, and kidney of both control and transgenic mice with a predominance of the -2S isoform. The human *CEACAM1* gene, in contrast, generates mRNAs with different splice isoforms, namely -4L/S and -3L/S. The expected human splice isoforms were observed in the BAC2-12 transgenic line, with a predominance of -4S in the liver and -4L in the intestine and kidney. Given the differences in murine and human splice isoforms observed in the transgenic animals, we conclude that the information governing the production of differentially spliced mRNA isoforms must be encoded in their respective genes. A comparison of the gene sequences at putative splice recognition sites may reveal which motifs are responsible for the observed mRNA species and tissue specific splicing. Preliminary studies on the human gene have begun to identify motifs responsible for generation of the long and short cytoplasmic domain splice forms [48]. The ratio of long and short CEACAM1 protein isoforms could critically affect cell function, since the short form lacks the ITIM motif. While the significance of the number of ectodomains is less well understood, the relative strength of cell-cell adhesion may be affected [2].

The expression of murine CEACAM1 in the developing embryo at both the message and protein level has been studied in detail [49]. Notably, murine CEACAM1 was found in the developing gut, lung, and kidney epithelium. However, the occurrence of two *Ceacam* genes in the mouse (and not in human) complicates the picture, with the *Ceacam2* gene mainly expressed in the kidney and testis [37]. The high expression of CEACAM1 in murine liver is striking and has been shown to regulate insulin clearance [39]. The pattern of human CEACAM1 expression closely mimics the mouse, with perhaps a caveat about the differential expression of the two murine *Ceacam* genes in the mouse. In our study of the human *CEACAM1* transgenic mouse, we were able to compare the staining of tissues for mouse and human CEACAM1. Except for the kidney, the tissue staining patterns and intensities were similar. In the case of the kidney, the patterns were identical but the intensity of staining for human CEACAM1 was greater than for murine CEACAM1. This result probably reflects the higher expression of murine *Ceacam2* (not measured) over that of *Ceacam1* in the kidney.

We were especially interested in the expression of the human gene in the immune system. Murine CEACAM1 is known to be expressed on spleen B-cells [50], serving as a receptor for the murine hepatitis virus. Similarly, human CEACAM1 is expressed on germinal center B-cells [17]. In the case of murine and human T-cells, little or no surface expression is observed on resting T-cells, but is highly expressed on activated T-cells [14], [51]. As expected, we found cell surface expression of both murine and human CEACAM1 on splenic B-cells, but not on splenic T-cells. Similar expression of murine and human CEACAM1 is expected on the surface of neutrophils, whether from the bone marrow or blood. Indeed, we found high levels of murine and human CEACAM1 on neutrophils from either source. Based on this preliminary analysis, it is likely that the CEACAM1 expression in the immune system mimics that found in mouse and man.

The model system was further validated by examining the binding of one of the Opa proteins found in *Neisseria* and known to bind to human CEACAM1. In this example, we chose Opa_52_ a *Neisseria* specific protein which had previously been shown to confer binding to human neutrophils or to Hela cells transfected with human CEACAM1 [8]. Since *Neisseria* may adhere to and be phagocytosed by neutrophils via a variety of receptors, we chose to demonstrate Opa_52_ binding using *E. coli* expressing this protein. As expected, the Opa_52_ positive bacteria were bound in greater numbers by the transgenic neutrophils than the Opa_52_ negative bacteria. Thus, the model appears to mimic what is observed with human neutrophils using Opa_52_ positive *Neisseria*. Furthermore, the model should allow investigation into insulin clearance, vasculogenesis, lumen formation, and other functions ascribed to human CEACAM1 in a physiological setting.
